# A soft artificial muscle driven robot with reinforcement learning

**DOI:** 10.1038/s41598-018-32757-9

**Published:** 2018-09-28

**Authors:** Tao Yang, Youhua Xiao, Zhen Zhang, Yiming Liang, Guorui Li, Mingqi Zhang, Shijian Li, Tuck-Whye Wong, Yong Wang, Tiefeng Li, Zhilong Huang

**Affiliations:** 1 State Key Laboratory of Fluid Power and Mechatronic Systems, Zhejiang University, Hangzhou, 310027 China; 2 Department of Engineering Mechanics, Zhejiang University, Hangzhou, 310027 China; 3 Key Laboratory of Soft Machines and Smart Devices of Zhejiang Province, Zhejiang University, Hangzhou, 310027 China; 4 Department of Chemical and Biological Engineering, Zhejiang University, Hangzhou, 310027 China; 5 Department of Computer Science, Zhejiang University, Hangzhou, 310027 China; 6 Advanced Membrane Technology Research Centre, Universiti Tekonologi Malaysia, Johor, 81310 Malaysia

## Abstract

Soft robots driven by stimuli-responsive materials have their own unique advantages over traditional rigid robots such as large actuation, light weight, good flexibility and biocompatibility. However, the large actuation of soft robots inherently co-exists with difficulty in control with high precision. This article presents a soft artificial muscle driven robot mimicking cuttlefish with a fully integrated on-board system including power supply and wireless communication system. Without any motors, the movements of the cuttlefish robot are solely actuated by dielectric elastomer which exhibits muscle-like properties including large deformation and high energy density. Reinforcement learning is used to optimize the control strategy of the cuttlefish robot instead of manual adjustment. From scratch, the swimming speed of the robot is enhanced by 91% with reinforcement learning, reaching to 21 mm/s (0.38 body length per second). The design principle behind the structure and the control of the robot can be potentially useful in guiding device designs for demanding applications such as flexible devices and soft robots.

## Introduction

Conventional robots are made of rigid components to provide large output force, high precision, and ease of controllability. When operating in complex environments, bio-inspired soft robots possess unique advantages^1–4^. Natural creatures are adaptive and resilient to environment. Fabricated with soft and deformable polymers, bio-inspired robots that mimic natural creatures have drawn a growing interest in recent years. Ultimately, soft robots can perform various tasks beyond the limits of conventional robots, achieving instinctive characteristics in terms of safe for humans^1^, geometric adaptation^4^, and tunable camouflage^5^.

Unmanned underwater vehicles play a significant role in engineering machines which can execute various missions, such as the study of marine life, investigation of underwater creatures, and exploration of the sea^6^. However, conventional unmanned underwater vehicles are less adaptive to environments, and they also create unwanted noise during the mission. These shortcomings undoubtedly reduce their utility. Here, soft robots based undersea vehicles are potential substitutes to work in the complex ocean environment. Various kinds of soft stimuli-responsive materials have been used to drive soft aquatic robots, such as dielectric elastomer, shape memory alloy^7^, ionic polymer metal composites^8^, and ionic conducting polymer films^9^. Among them, dielectric elastomer (DE) has stood out due to its exceptional fast response and large actuation^10–13^. Utilizing DE as base structure, a jellyfish^14^, a Manta-ray^15^, and a soft swim-bladder robot^16^ have been designed recently.

Currently, the most widely used approaches to control the robot are assuming the mechanical structure as rigid body, but those approaches are not applicable on soft robots. In general, the actuation of soft robot itself is difficult to be modelled^17^. Even for a simple task, it usually requires complicated mechanical analysis^18,19^. To-date, there are significant contributions by experts of artificial intelligence (AI) and robotics in trying to surmount the modeling and learn to perform specific tasks for soft gripper based on imitating and reinforcement learning^20,21^. Reinforcement learning (RL) is an adaptive control strategy that serves as a potential solution to the control of soft robots.

Unlike fishes that acquire thrust most often by wave-like movements of the fish’s body, fins and tail, cuttlefish and jellyfish move by jet propulsion. In detail, these cephalopods draw water into their body, then expel the jet of water from a rear orifice to generate a series of vortex rings and hence thrust. This mechanism has been studied in-depth by researchers^22,23^. Inspired by the structure and propulsion mechanism of cuttlefish, we have designed a biomimetic cuttlefish robot with DE membranes (3 M VHB) as the artificial muscles. The cuttlefish robot uses surrounding open water as the electric ground^15^, which makes it more robust when faced with the insurmountable challenge of high voltage to actuate the artificial muscle (DE membrane). To make the cuttlefish robot more compact, a highly compact electric system (Epod) is selected for both remote control and voltage boosting. Other than design of the overall on-board system, reinforcement learning is implemented to optimize the strategy toward the actuation of the cuttlefish. Finally, the cuttlefish robot reaches to a swimming speed of 21 mm/s, much better than the one without learning.

**Figure 1:**
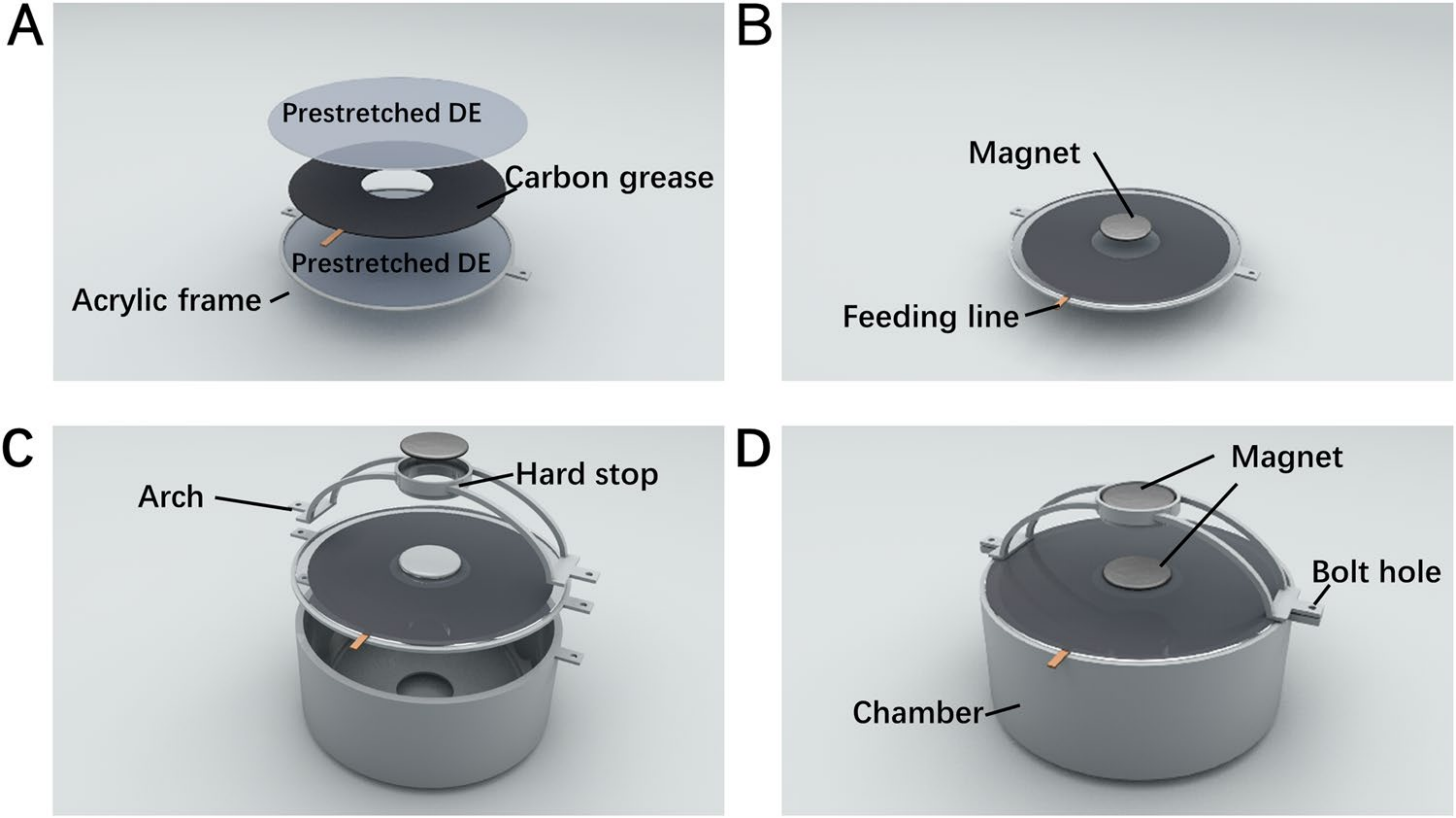
Fabricating the jet-actuator of the robot. (**A**) Fabrication of muscle laminate: A DE membrane (VHB membrane with initial thickness of 1 mm) was biaxially pre-stretched (3 × 3). Carbon grease was sandwiched with the shape of rings (inner diameter 25 mm, outer diameter 75 mm) by two pre-stretched membranes. (**B**) Two circular magnets with diameter of 20 mm was put on the center of both sides of muscle laminate. (**C**) Muscle laminate was assembled on the chamber. (**D**) An arch was assembled with a magnet on the top of the acrylic chamber.

Figure 1 shows the detailed fabrication process of the jet-actuator of the robot. The artificial muscle laminate consists of a thin circular layer of carbon grease sandwiched between two pre-stretched DE membranes, along with a small piece of thin foil as the electric feeding line. The chamber is made of acrylic with diameter of 95 mm and height of 30 mm. There is an orifice with diameter of 20 mm at the bottom of the acrylic chamber. The arch with height of 25 mm is used to place the permanent magnet (PM) made of Neodymium, so it can provide the attractive force and the pre-stretched muscle will bend into a cone-like shape. The hard stop is used to prevent excessive attractive force of magnets. The height of body is 55 mm. In consideration of the weight of high voltage (HV) supply and wireless communication system, the total weight of onboard system is still relatively light which is only 126 g. Bolt holes are used for plastic screws to tune the initial distance *d3* between the magnets, which is found to have a significant influence on the overall performance of the cuttlefish robot.

**Figure 2:**
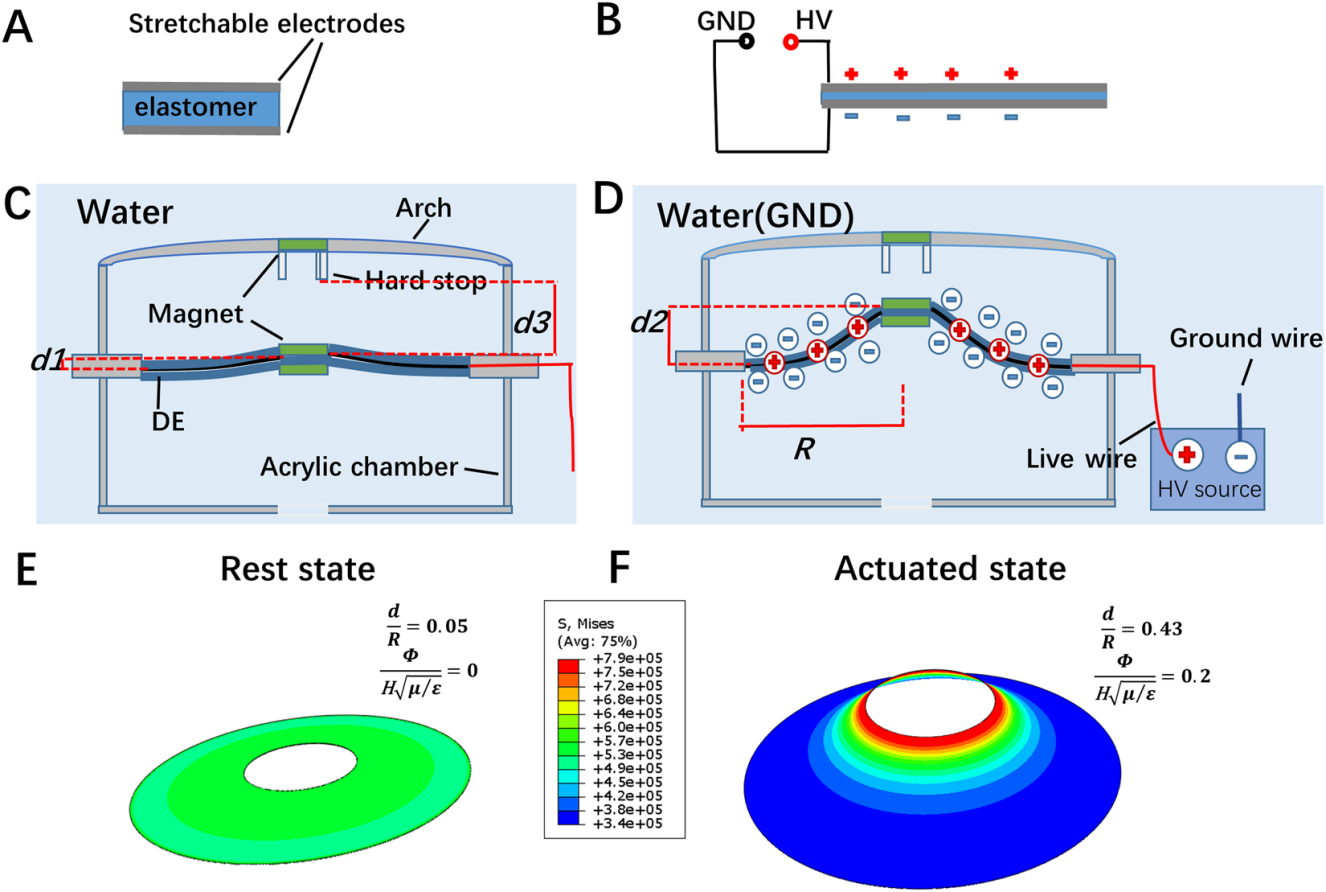
Operating mechanism of the cuttlefish robot. (**A**) DE membrane serves as flexible capacitors with stretchable electrodes on both sides of the DE membrane. (**B**) When a high voltage is applied on one side of the DE membrane (another side is served as electric ground), Maxwell stress will start to act on the electrodes and cause the reduction in the thickness of the DE membrane, resulting the expansion of area due to the incompressibility of DE. (**C**) Rest state of jet-actuator with initial displacement *d1* due to the attractive force of magnets. (**D**) Actuated state of the jet-actuator with displacement *d2* (surrounding water as electric ground). The expansion of area due to the application of high voltage on the muscle laminates results in relaxing the pre-stretched membrane and then the reduction of the stiffness parallel to thickness. (**E**) Tilted view of the FEA simulation for the reset state of the cuttlefish robot with no voltage. (**F**) Tilted view of the FEA simulation of the actuated state of the cuttlefish robot with dimensionless voltage of 0.2.

Figure 2 shows the actuation mechanism of the cuttlefish robot. DE based flexible capacitor can reduce its thickness with the application of high voltage. The thickness reduction is caused by Maxwell stress when positive and negative charges respectively accumulated on each sides of the DE membranes. Due to the incompressibility of DE material, the surface area of the capacitor will expand (Fig. 2A,B). For the jet-actuator, magnets based mechanical biasing mechanism is used to enlarge the displacement^24^. When no voltage is applied on the pre-stretched DE membrane (rest state), the initial displacement *d1* due to the attractive force of the magnets is relatively small (Fig. 2C). When high voltage is applied, the pre-stretched DE membrane is relaxed, decreasing its stiffness in the axial direction of the jet-actuator. After the relaxation, the displacement *d2* is much larger than initial displacement *d1* (Fig. 2D). The actuation of the DE membrane induces the volume change of the chamber, resulting in the jet-refill cycle of the cuttlefish body, and generating propulsion to drives the robot. To further investigate the actuation of DE membrane, we simulate the structural deformation through finite element analysis (FEA). In the analysis process, the dimensionless voltage represents the voltage applied on the jet-actuator where Φ is the applied voltage, μ is the shear modulus of the material, ε is the permittivity and H is the initial thickness. Besides the applied voltage, dimensionless displacement load is also imposed in FEA, where *R* is the radius of the membrane and *d* is the axial displacement. A material model from a previous study^25^ was embedded into Abaqus with the user-defined subroutine UMAT. As a result, the von Mises stress distribution corresponding to the rest and actuated state are shown (Fig. 2E,F). The stress distribution reveals the inhomogeneous deformation of the actuator, indicating the use of the material is not efficient^26^. Some regions of the membrane are near to the failure whereas others are still far below the limit. We foresee that inefficient use of the material can be solved by variable thickness of the membrane which demand further research.

## Results

**Figure 3:**
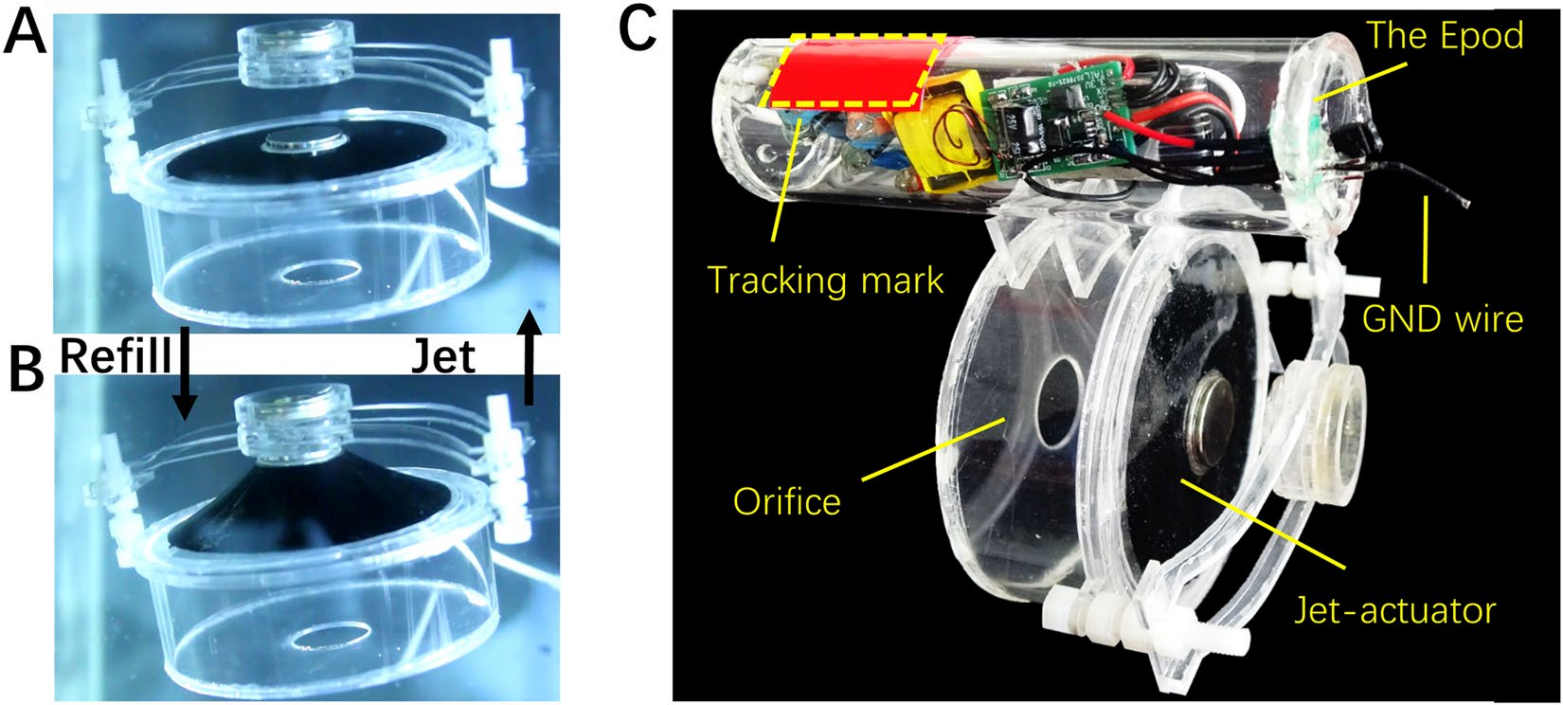
(**A**) The rest state of the jet-actuator. (**B**) The actuated state of the jet-actuator. (**C**) The schematic of the cuttlefish robot.

We aim to design an untethered cuttlefish robot with onboard system providing power and control. The initial distance *d3* between magnets is 15 mm and the initial displacement *d1* of the jet-actuator is set at 2 mm (Fig. 3A). When applying a voltage of 6.8 kV (charged), the recorded displacement *d2* is 17 mm and water is drawn into the chamber (Fig. 3B). As soon as it is discharged, the water is expelled out through orifice. With such reversible motion of the artificial muscle, thrust is thus generated to propel the cuttlefish. Accordingly, the simple in-plane DE actuation is transformed into periodical volume change of the chamber, similar to the working principle of motor transmission system but in a more compact form. Figure 3C shows the system of the robot, including the compact high voltage source, the battery and the jet-actuator. The red area is the tracking mark, indicating the location of the robot in real time. Movement of the robot is voltage dependent as it is very much depending on the jet of water^7,14^. RL is used to optimize the actuation pattern in order to enhance the performance of our robots, and the details will be addressed in later section. The Epod (powered by a 3.7 V lithium-ion battery) is sealed in an acrylic tube to provide enough buoyancy for the cuttlefish. It is controlled by an eight-pin microcontroller unit (MCU) and the output voltage amplitude (0 V to 10 kV) is adjusted by pulse-width modulation duty cycle. 2.4 G ZigBee is attached on the Epod which enables wireless control with a computer. More details of the Epod can be found in our previous works^15,16^. The length of the tube is 110 mm with diameter of 35 mm.

**Figure 4:**
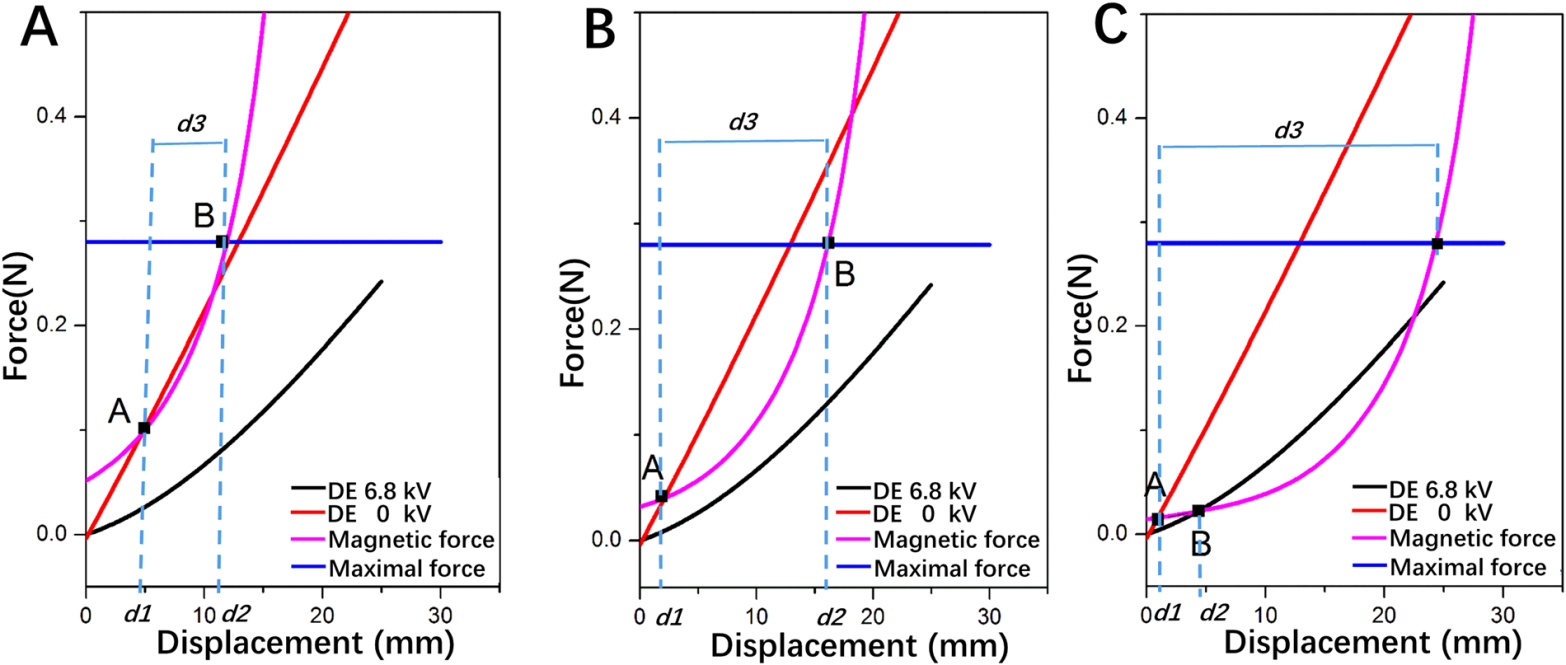
The relation of the force and displacement for various initial distance *d3* measured by uniaxial tensile machine. (**A**) *d3* = 6 mm. (**B**) *d3* = 15 mm. (**C**) *d3* = 24 mm

Previous works have been reported to compare the displacement achieved by different kinds of biasing mechanisms such as hanging masses, springs, permanent magnets, etc.^24^. Inspired by those works, experiment has been performed to evaluate the influence of initial distance *d3* on the performance of actuator (Fig. 4). Force and displacement curves of the jet-actuator actuated with 6.8 kV and 0 kV (without magnets) are recorded and plotted for comparison. Besides, Magnetic force (the biasing force) and displacement curve are also recorded. The influence is justified by investigating the intersection of the biasing force-displacement curve with DE curves. Generally, when applying a voltage of 6.8 kV (charged), the DE curve shifts indicating the decrement in stiffness. In Fig. 4, the point marked with “A” is the equilibrium point when no voltage is applied, while the point marked with “B” is equilibrium point under high voltage. The maximal force of attraction that the magnets could provide is constant for various *d3* since the length of the hard stop is set constant. The initial distance *d3* is set as 6 mm in Fig. 4A. Due to the higher force of magnets, the DE curves of 6.8 kV will be attracted to the hard stop (corresponds to stage B). When the voltage is cut-off, the DE curves of 0 kV is lower than magnetic force curve at stage B (means the actuator remains at stage B). In this condition, stable reversible motion can’t be achieved, so we infer that initial distance of magnets should not be too small. The initial distance *d3* is set as 15 mm in Fig. 4B. It shows that the DE curves of 0 kV is above magnetic force curve at stage B and thus stable reversible motion can be achieved. In this case, actuator can be pulled back from stage B to stage A where the recorded reversible stroke is 15 mm. When the initial distance *d3* is set as 24 mm, the reversible motion can be observed and the recorded displacement between stage A and B is relatively small, just 3 mm (Fig. 4C). It results in small volume change of chamber. The three initial distances of magnets correspond to three typical behaviors. However, the quasi-static modeling discussed above doesn’t fully reflect the actual influence of the discharge rate of Epod and actuation frequency on the performance of the cuttlefish robot. Besides, slight variation in *d3* significantly affects the generation of reversible motion. As a result, adaptive control method is required to enhance the actuation of DE membrane and the propulsion of the robot. In order to generate relatively large volume jet of water and reversible motion, the initial distance *d3* is fixed at 15 mm, which corresponds to force-displacement relation of the jet-actuator in Fig. 4B.

Traditional actuators^27^, such as electric motors or pumps, are in the mainstream of robots controlling research in comparison with DE actuators. As part of the objective in this study, actuation patterns are crucial to the robot in order to swim fast. For soft actuator-based robots (SARs), researchers usually tune the frequency and amplitude of actuation manually^7,15^. Currently, there is no reliable method to enhance the locomotion of SARs, *i*.*e*. moving velocity of the robot. In this work, we propose the use of reinforcement learning to address this problem. Generally, RL enables a robot to autonomously discover an optimal policy to maximize cumulative reward through trial-and-error interaction with its environment^28^. RL tends to solve the problem based on the assumption of Markov decision processes (MDPs) which consist of a set of states *S*, a set of actions *A*, the rewards *R*, and transitions *T*. Therefore, how to choose states that can reflect the actual characteristics of the SARs is quite challenging. At first, trade-off must be considered. Continuous states and actions could fully explore the potential of SARs, but they will make state the space and action space too large to be solved. Since we use the voltage to actuate the robot, discretization can be effective for low dimensional problem^27^, thus we discretize the action, *i*.*e*. only two kinds of voltage amplitude (0 kV and 6.8 kV) within unit time are used in our experiments. Undeniably, states which include the nature of the robot and hydrodynamics is extraordinarily complicated. To make RL algorithm easier to be implemented, we choose the several actions (*k* times) as the state.

$$\begin{array}{rcl}{s}_{t} & = & \{{a}_{t-k},{a}_{t-k+1},{a}_{t-k+2},\,\ldots \,{a}_{t-1}\}\,{\rm{for}}\,{\rm{last}}\,k\,{\rm{actions}}\\ {a}_{t} & = & 0\,{\rm{kV}}\,{\rm{or}}\,6.8\,{\rm{kV}}\end{array}$$

According to the experiments, such simplification can enhance the performance of the robot. The reward function depends on the task that we are trying to complete for RL tends to maximize the cumulative rewards. To maximize the velocity, we define the reward function *r*_t_ as:

1 $${r}_{t}=displacement(t+1)-displacement(t)$$

**Figure 5:**
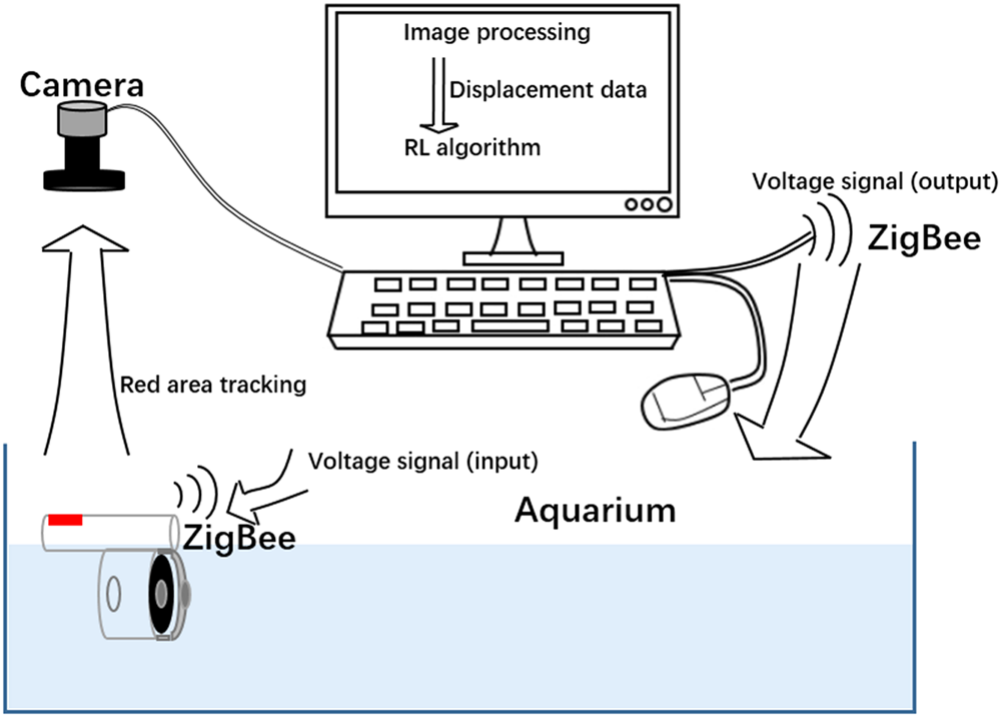
Schematic diagram of the experiment setup for the cuttlefish robot.

Second, in terms of RL algorithm, we use the Q-learning^29^ with an experience replay mechanism in which we store the agent’s experiences at each time-step, *e*_t_ = (*s*_t_, *a*_t_, *r*_t_, *s*_t+1_) in a data-set D = *e*_1_, …, *e*_N_, pooled over many episodes into a replay memory. Details of the algorithm are shown below. The displacement data is acquired by processing the image from a camera. The reward can be calculated from eq. (1) using the displacement data. And action generated from the RL algorithm is the voltage signal which transfers from the computer to the cuttlefish robot via ZigBee. The experiment setup for the cuttlefish robot is illustrated in Fig. 5. Due to the limitation of camera processing rate, we can only sample 20 frames per second. We chose unit time as 0.2 s for RL to generate an action of 0 kV or 6.8 kV through the computer. *k* = 6 is a relatively suitable choice for the RL, since jet-actuator can perform a full jet-refill cycle within 6 unit time.

**Algorithm:**
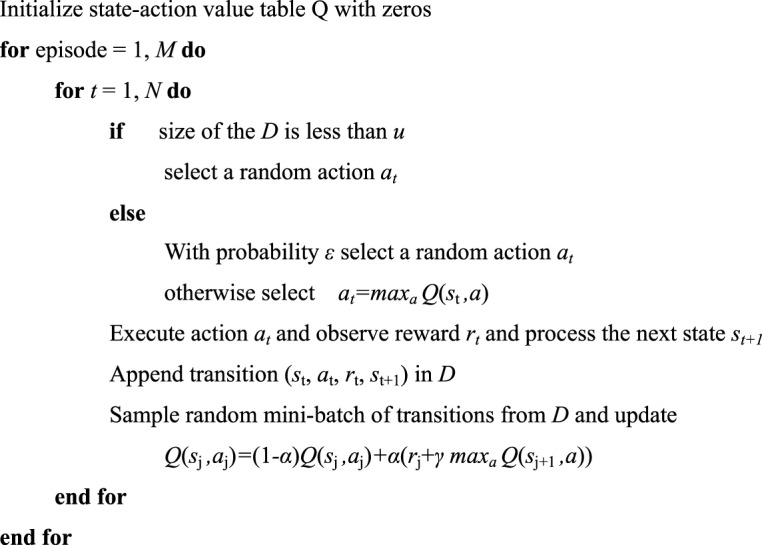
Q-learning with Experience Replay.

### The algorithm

**Figure 6:**
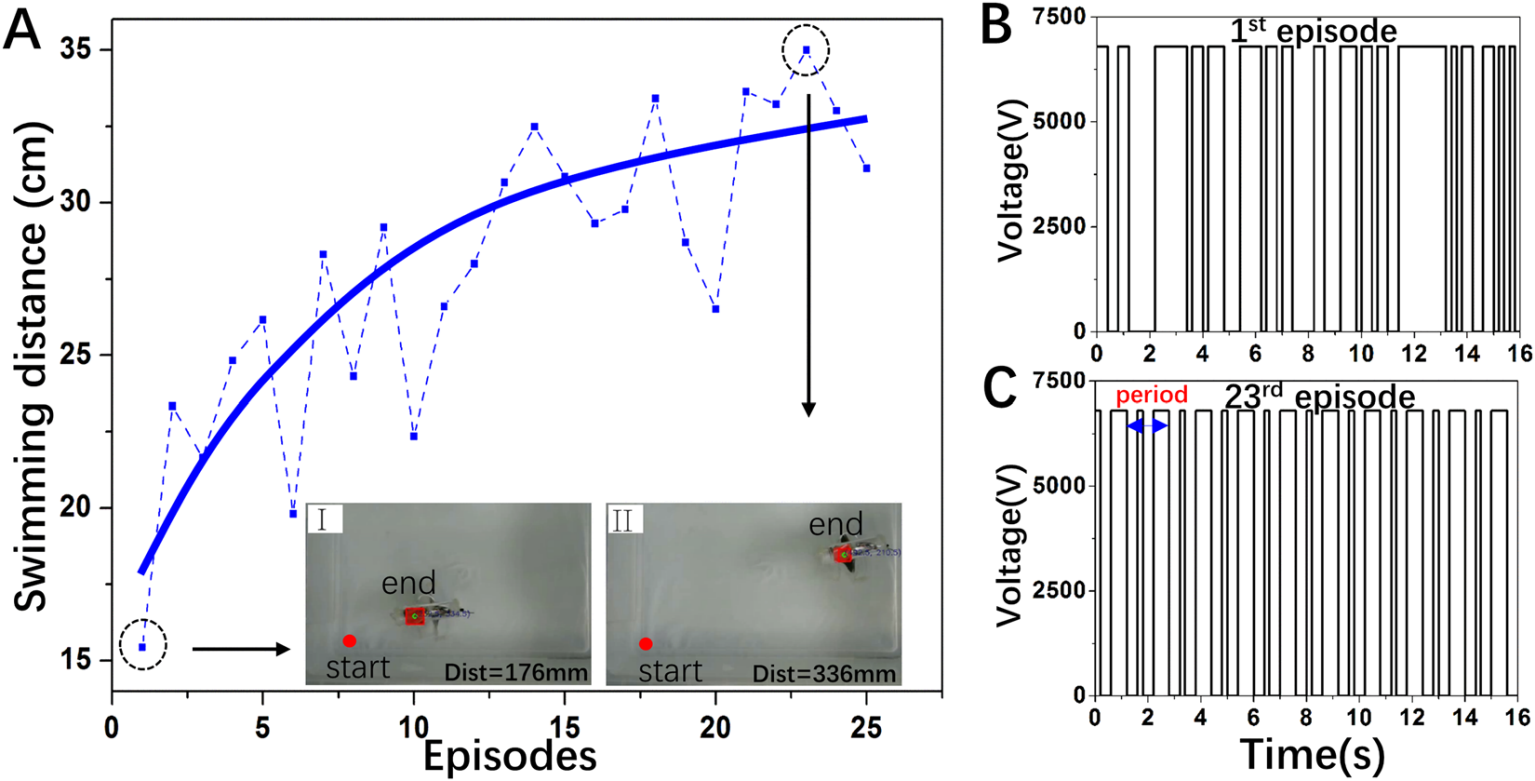
(**A**)The total displacement within 16 seconds for each episode. (**I**) Snapshot of the cuttlefish robot of the 1^st^ episode. (**II**) Snapshot of the cuttlefish robot of the 23^rd^ episode. (**B**) Sequence of actions of the 1st episode. (**C**) Sequence of actions of the 23rdst episode.

Table *Q*, state-action value table, is the guidance to give an action. At first, we initialize it with zeros. After each unit time, we will get a transition (s_t_, a_t_, r_t_, s_t + 1_), and we will append it in D. We use equation (2), also called backward induction^30^, to update table Q with a mini-batch of transitions uniformly sampled from D, which is the core of the algorithm. This is based on the following intuition that the optimal strategy is to select the action *a*_t_ maximizing the expected value of *r*_t_ + *γQ*(*s*_t+1_,*a*_t+1_). *α*, the learning rate, determines to what extent newly acquired information overrides old information. The discount factor *γ* determines the importance of future rewards, and ensures that the state-action value be finite when updating table *Q*. when the robot is in state *s*_*t*_, the algorithm chooses to give action randomly with probability *ε*, or it will give action which has maximal *Q* value at *s*_*t*_. To ensure the results are comparable, number of actions *N* is fixed as 80 during each episode. Besides, there is a threshod size *u* of *D* which guarantees the diversity of the experience before leaning. *u*, α and *γ* are set as 200, 0.1 and 0.9 respectively. As soon as an episode completed, cuttlefish is placed static at the start point before we proceed the next episode. With decreasing *ε*, we rely more on the value table *Q* to choose actions, which indicates experience gradually been exploited. The results are shown in Fig. 6.

In total 25 episodes of the RL process, the swimming performance of the cuttlefish robot constantly rises with fluctuation (Fig. 6A). Figure 6A(I) shows that the robot swims with the distance of 176 mm in 16 seconds. The average speed of the cuttlefish robot in the 1st episode is 11 mm/s (0.2 body length per second). Figure 6A(II) shows that the robot swims with the distance of 336 mm in 16 seconds. The average speed of the cuttlefish robot in the 23^rd^ episode is 21 mm/s (0.38 body length per second), which is 91% faster than that of the 1^st^ episode (see Supplementary Movie). The sequence of driving voltage of the 1^st^ episode is relatively chaotic (Fig. 6B). With the process of RL, the sequence of driving voltage gradually converges with periodic pattern (Fig. 6C). The experimental results demonstrate that the robot can autonomously actuate DE membranes with optimized control by RL, enhancing the swimming performance.

## Discussion

In summary, we have designed a cuttlefish robot with DE as the jet-actuator. The surrounding water functions as the highly robust electrode of the ground end. We have showed that the high voltage required DE system is compatible with the aqueous operating environment. The chamber and magnets are interacted with the actuating DE membrane to function as jet-actuator that converts the in-plane actuation of DE membrane into the propulsion with the jet-refill cycles. The excellent actuation of the DE membrane, when combined with integrated compact electronics for power and remote control, results in successful operation of an untethered cuttlefish robot. Furthermore, we have investigated the influence of initial distance between magnets on the deformation of the jet-actuator. RL is used to optimize the actuation strategy, enhancing the swimming performance of the robot. The swimming speed of the robot is enhanced by 91% with reinforcement learning, reaching to 21 mm/s (0.38 body length per second). Although the swimming behaviors of the robotic cuttlefish fluctuates due to the complexity of the RL process, actuation motion and the hydrodynamic drag, the average speed of the robot keeps rising. The experimental results validate that the optimized control by RL can enhance the actuation performance of DE driven soft actuator-based robots. The robot can’t change its direction at present, but we foresee that the direction could be changed by using another soft actuator to adjust direction of the jet. Overall, all these performance features are highly desirable for soft robots driven by various types of soft artificial muscle. And the mechanical structure and RL strategy design principle of our robot can be potentially useful in guiding device designs for demanding applications such as flexible devices and soft robots.

## Methods

The DE membranes (initial thickness, 1 mm) was made from 3M VHB4910 membrane. Silicone adhesive glue (Dow Corning 734) was used to seal the intersection of the feed line. The finite element analysis was using Abaqus 6.13. Hybrid, reduced integration elements (CAX4RH) were used in the simulation. Tacking of the cuttlefish robot was based on OpenCV^31^ library with a Logitech camera C270. The permanent magnet was made of Neodymium.

## Electronic supplementary material

Swimming of the robot in the 1st and the 23rd episode.

Supplementary Dataset 1

## Data Availability

All data needed to evaluate the conclusions in the paper are present in the paper and/or the Supplementary Materials. Additional data related to this paper may be requested from the authors.
